# *Saccharomyces cerevisiae* morphological changes and cytokinesis arrest elicited by hypoxia during scale-up for production of therapeutic recombinant proteins

**DOI:** 10.1186/s12934-018-1044-2

**Published:** 2018-12-20

**Authors:** Juan C. Aon, Ricardo C. Tecson, Vakhtang Loladze

**Affiliations:** 1 Department of Microbial and Cell Culture Development, Research and Development, GlaxoSmithKline, 709 Swedeland Road, King of Prussia, PA 19406 USA; 2 Department of Bioanalytical Sciences, Research and Development, GlaxoSmithKline, 709 Swedeland Road, King of Prussia, PA 19406 USA

**Keywords:** *Saccharomyces cerevisiae*, Fermentation bioprocess, Scale-up, Hypoxia, Mitosis, Cytokinesis, Metabolomics, Cell wall

## Abstract

**Background:**

Scaling up of bioprocesses represents a crucial step in the industrial production of biologicals. However, our knowledge about the impact of scale-up on the organism’s physiology and function is still incomplete. Our previous studies have suggested the existence of morphological changes during the scale-up of a yeast (*Saccharomyces cerevisiae*) fermentation process as inferred from the volume fraction occupied by yeast cells and exometabolomics analyses. In the current study, we noticed cell morphology changes during scale-up of a yeast fermentation process from bench (10 L) to industrial scale (10,000 L). We hypothesized that hypoxia observed during scale-up partially impaired the availability of *N*-acetyl-glucosamine, a precursor of chitin synthesis, a key polysaccharide component of yeast mother-daughter neck formation.

**Results:**

Using a combination of flow cytometry with two high throughput cell imaging technologies, Vi-CELL and Flow Imaging, we found changes in the distribution of cell size and morphology as a function of process duration at the industrial scale of the production process. At the end of run, concomitantly with lowest levels of dissolved oxygen (DO), we detected an increase in cell subpopulations exhibiting low aspect ratio corresponding to morphologies exhibited by large-single-budded and multi-budded cells, reflecting incomplete cytokinesis at the M phase of the yeast mitotic cycle. Metabolomics from the intracellular milieu pointed to an impaired supply of precursors for chitin biosynthesis likely affecting the septum formation between mother and daughter and cytokinesis. Inducing hypoxia at the 10 L bench scale by varying DO levels, confirmed the existence and impact of hypoxic conditions on yeast cell size and morphology observed at the industrial scale.

**Conclusions:**

We conclude that the observed increments in wet cell weight at the industrial scale correspond to morphological changes characterized by the large diameter and low aspect ratio exhibited by cell subpopulations comprising large single-budded and multi-budded cells. These changes are consistent with impairment of cytokinesis triggered by hypoxia as indicated by experiments mimicking this condition at DO 5% and 10 L scale. Mechanistically, hypoxia impairs *N*-acetyl-glucosamine availability, a key precursor of chitin synthesis.

## Background

We reported physiological changes in the yeast *Saccharomyces cerevisiae* induced by hypoxia when a high-cell density fed-batch production process was scaled-up by 1000-fold [1]. We hypothesized that low oxygen availability at the 10,000 L scale changed the physiological state affecting yeast growth, while increased cell volume suggested compromised cell wall integrity [2]. These changes induced modifications in cell morphology, which in yeast is determined by the cell wall, a rigid structure that provides mechanical protection, dictates cell shape, and modulates the selective uptake of macromolecules throughout the different stages of the yeast’s life cycle [3–5].

Substantial changes in the composition and structure of the cell wall take place during bud emergence and growth, septum formation, and cell separation. A new bud essentially lacks chitin, and mannoprotein expression is different from that of a mature mother cell. Such variations determine the permeability of the cell wall, which increases in the initial stages of the budding process [6]. The chemical composition of the cell wall is apparently uniform around the ellipsoidal cell except for the septum and the chitin ring that encircles it, which shows a high chitin-to-glucan ratio [3]. During the cell cycle the diameter of the mother-bud neck, where the chitin ring and the primary septum arise, remains the same. Therefore, some mechanism must limit growth to the bud and block it at the boundary between mother and daughter cell. The importance of this growth control is obvious, because in *S. cerevisiae*, unlike fission in yeast and animal cells, the site for cytokinesis is created and partially organized at the site of the bud emergence [7]. Consequently, it appears that a strict regulation of the balance between synthesis and degradation pathways for the cell wall polymers, as well as secretion of enzymes and cell wall components, is critical [8]. All these processes respond to cell cycle controls as well as to environmental signals such as availability of nutrients and oxygen [9, 10].

Three different chitin synthases (CSI, CSII, and CSIII) with defined roles are involved in the formation of the mother-bud neck during the yeast life cycle, and their activities are regulated by specific spatio-temporal mechanisms. CSI is a membrane-bound enzyme that catalyzes the transfer of *N*-acetyl-glucosamine (GlcNAc) residues from UDP-GlcNAc to a growing chain of chitin, a polysaccharide of β-(1,4) linked GlcNAc (2-acetamino-2-deoxy-β-d-glucose). CSI and CSII appear to be necessary only at a specific time and at a precise site during the mitotic cycle [11]. In contrast, chitin synthesis by CSIII occurs at several locations during the yeast life cycle.

Previous data showed the cell wall formation biosynthesis pathways which are affected by hypoxia in 10,000 L bioreactors. Aon and colleagues [2] reported that at the 10,000 L scale cells were exposed to more severe hypoxia triggering impairment in oxidative phosphorylation and the synthesis of precursors such as GlcNAc. It appears as though, we reported the involvement of chitin synthesis and formation of proper primary septum during cytokinesis [12], likely eliciting the arrest of cell proliferation.

In recent work, focus was placed on characterizing the yeast morphological changes and understanding the underlying metabolic mechanisms leading to those modifications triggered by hypoxia at the industrial production scale. The morphology of different cell subpopulations and their distribution in the different phases of the yeast mitotic life cycle at different levels of dissolved oxygen are described using a proven small scale model (SSM) of the production process [13, 14]. From a metabolic perspective, we interpret our results in terms of the reduced availability of precursors triggered by hypoxia resulted in impaired cell wall formation, lack of integrity and functionality.

## Results

### Biomass accumulation affected by dissolved oxygen availability

We previously reported [1, 2] a clear increase in the volume occupied by yeast cells in 10,000 L bioreactors, as revealed by wet cell weight (WCW) measurements. It was postulated that hypoxia may have played a role in the observation of increased wet cell weight, the impaired supply of intermediates for \cell wall formation as well as the release of intracellular metabolites key for biomass synthesis. To further investigate the role of hypoxia, hypoxia conditions were simulated in 10 L reactors by modulating oxygen availability (Table 1). We utilized the DO set point of 25% at 10,000 L as a reference because it corresponds to the successful scale-up of the bioprocess at the industrial production scale [1]. Bioreactors at the 10 L scale were set up at three different DO set points 12.5%, 8.5%, and 5%. All other scale-independent bioprocess parameters, including media composition and feed rates were kept constant. The condition at DO 5% was established after determining that the condition at DO 2% elicited a shift to respiro-fermentative metabolism throughout the entire duration of the production process, which did not allow the process to stay in state of control (data not shown).

**Table 1:** Study design and sampling

Scale (L) Number of batches per DO set point	Dissolved oxygen (DO) Set point (%)	EFTs of samples (h)
10,000 L (n = 3)	25.0	46.5, 54.0, 64.5, 76.5, 82.0
10 L (n = 3)	12.5	46.5, 54.0, 63.5, 76.0, 81.5
	8.5	
	5.0	

**Fig. 1:**
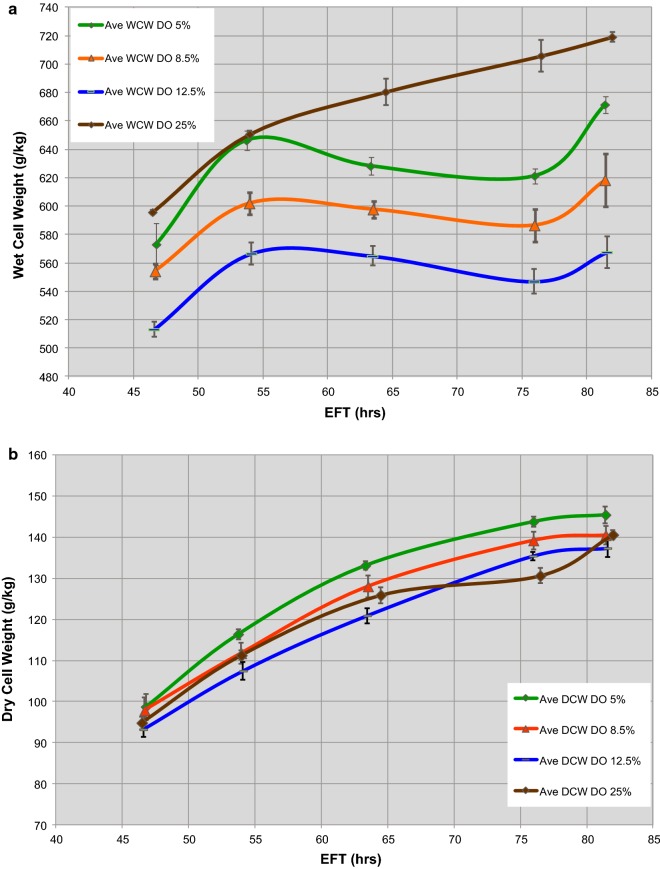
Comparison of time-course profiles of wet cell weights (**a**) and dry cell weights (**b**) of fermentations performed at 10,000 L scale and DO 25% (brown lines), and at 10 L scale set at three different DO set points, 5% (green lines), 8.5% (oranges lines), and 12.5% (blue lines). Plotted DCW, WCW values are average of triplicates at 10,000 L scale, and at 10 L scale per DO set point experimented. Error bars represented one standard deviation

Table 1 summarizes the sampling of the 10,000 L and 10 L scale cultures in triplicates at every DO set points and elapsed fermentation time (EFT). As shown by Fig. 1a, the 10,000 L scale WCW increased throughout the EFT span, there was quick increase from 47 to 54 h EFT but with a lower rate of increase from 54 h EFT until the end of the fermentation at 82 h EFT. The 10 L scale exhibited comparable profiles in time but not in the absolute values of WCW at the three different DO set points. Every profile showed similar slopes of increasing WCWs until 54 h EFT followed by decreasing WCWs until 76 h EFT to finally rise from 76 to 82 h EFT (Fig. 1a). As the DO was set to lower percentages (from 12.5 to 5%) at the 10 L scale, the WCW throughout the EFT span exhibited higher values, thus approaching the profile of WCWs observed in 10,000 L cultures. In addition, within the same EFT range, the dry cell weight (DCW) exhibited similar profiles at both scales and different DO set points, without a clear correlation between both parameters (Fig. 1b). Table 2 displays the values of WCW/DCW ratio indicating higher values in 10,000 L vs. 10 L, with comparable viability. At the end of run (EOR) in 10,000 L cultures, cells exhibited higher ratios compared to those at 10 L scale and DO set points of 12.5%, 8.5%, and 5%, with differences being 24%, 16%, and 11%, respectively.

**Table 2:** Final process-response measurements at every experimentally designed condition

EOR measurements	10,000 L (n = 6)	10 L (n = 6)		
	DO 25%	DO 12.5%	DO 8.5%	DO 5.0%
DCW (g/kg)	140.6	137.3	140.5	145.5
WCW (g/kg)	719.1	567.4	618.4	671.4
WCW/DCW ratio (± SEM)	5.12 (± 0.06)	4.13 (± 0.10)	4.40 (± 0.10)	4.62 (± 0.18)
Viability (%) (± SEM)	93.2 (± 1.1)	86.9 (± 2.0)	89.8 (± 1.0)	91.4 (± 0.5)

EOR stands for end of run, 82 h EFT. SEM means Standard Error of the Mean, DCW and WCW values are averages based on two determinations per time point (EFT) from three experiments

Collectively, these results suggest that an increase in cell volume occurs in the scale-up of the bioprocess. This increase in cell volume is hypothesize that it is due to the cultures experiencing hypoxia at the 10,000 L scale. We have attempted to validate this hypothesis by running the 10 L reactors at lower levels of DO set point. We hypothesize that this represents an adaptive physiological phenomenon of the yeast cell in response to experiencing conditions of hypoxia.

### Impact of scale-up and high cell density upon growth and the mitotic cycle of yeast

To investigate whether the yeast response represented an adaptive physiological phenomenon triggered by the existence of hypoxic pockets in large volume reactors, we assessed the impact of DO level on cell morphology, karyokinesis and cytokinesis to elucidate the relationship between cell volume, nuclear division and morphology. We were interested in whether the increase in cell volume at the 10,000 L scale mirrored yeast growth (i.e., net increase in cell mass due to cell division and/or increase just in cell size), and if so, which phase(s) of the yeast mitotic cycle were affected.

**Fig. 2:**
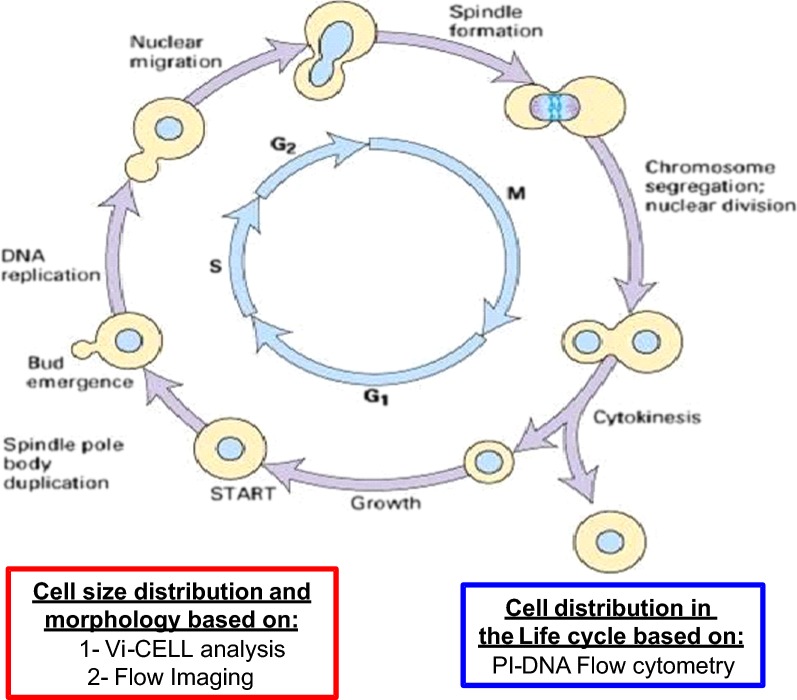
Diagram of *Saccharomyces cerevisiae* mitotic life cycle and its different phases. Morphologies and DNA distribution associated with every phase of the yeast vegetative growth. Technologies used in this study to reveal chromosome segregation/nuclear division based on propidium iodide-DNA (PI-DNA) staining and quantitation with flow cytometry; and cell size as well as morphology distribution based on Vi-CELL and flow imaging

Karyokinesis comprises the start of DNA replication in S phase followed by completion of nuclear division in M phase (Fig. 2) with cytokinesis occurring in late M phase. Two well-known control checkpoints based on critical size of daughter cells - START in late G1 phase and cytokinesis completion in late M phase, were also considered (Fig. 2) [15].

Yeast cells, sampled at the EFTs shown in Table 1, were stained with propidium iodide (PI) and their distribution quantified as subpopulations with one (1C), two (2C), three (3C), or in transition, sets of chromosomes using flow cytometry. Figure 1a and Table 2 showed that DO 5% at 10 L scale exhibited the closest WCW and WCW/DCW ratio at EOR to those determined at 10,000 L scale. Therefore, samples from the DO 5% batches were used for this study.

**Fig. 3:**
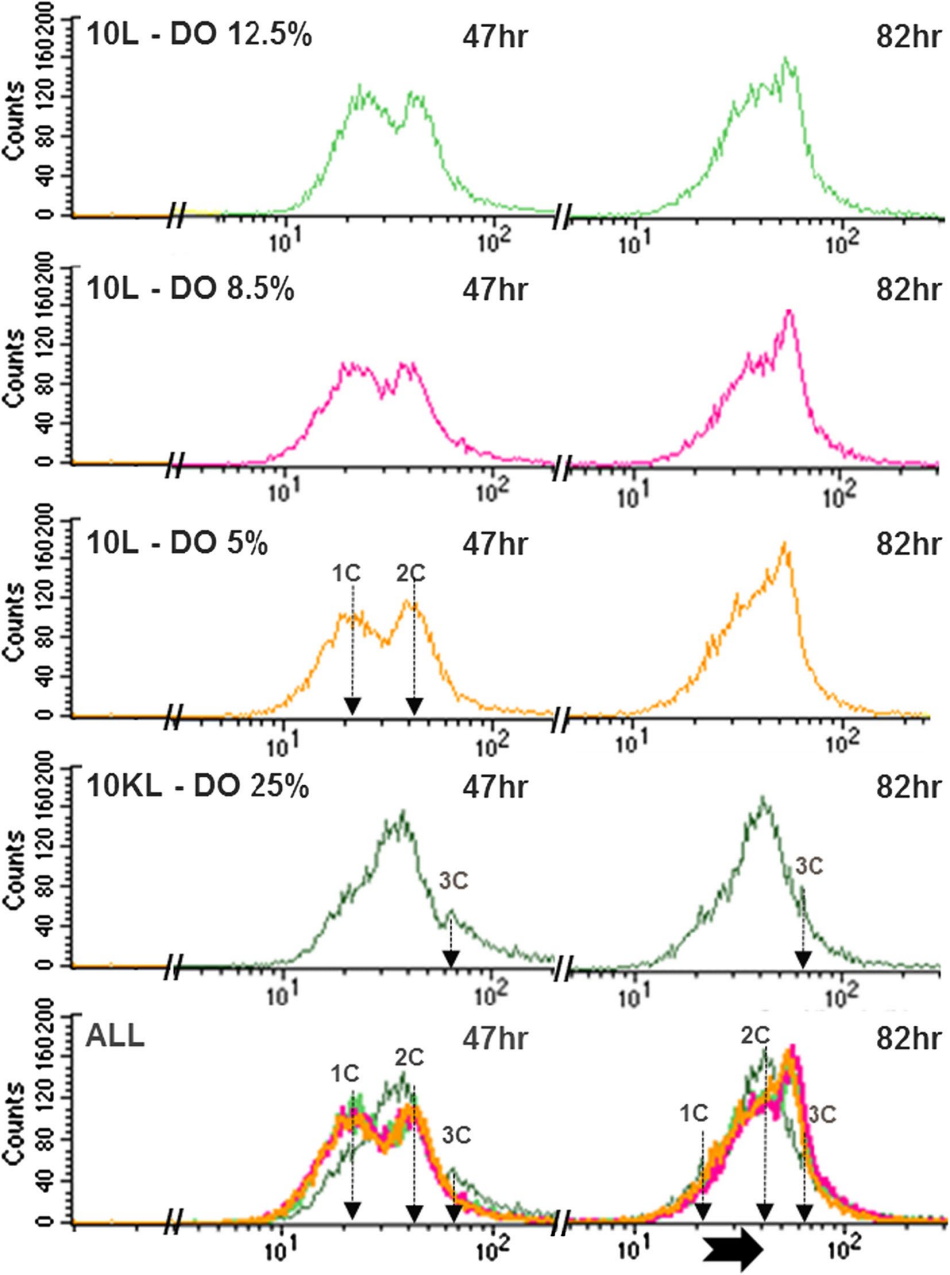
Quantification of cells with different DNA content by flow cytometry of fermentation at 10,000 L (10 KL) scale (dark green) and 10 L scale, within 10 L at DO 5% (orange), 8.5% (magenta), 12.5% (light green). Plots of counts of propidium iodide (PI) stained cells versus intensity of PI fluorescence at 47 (left hand-side profiles) and 82 hour (h) (right hand-side profiles) elapsed fermentation time (EFT). The DNA content is characterized by one set of chromosomes (1C), two sets (2C), up to three sets (3C) (shown by vertical arrows). For comparative purposes, all profiles at 47 h EFT were superimposed as well as those but separately at 82 h EFT. Purposely those overlays at both time points show the relative change of the subpopulations with different DNA content (1 set, 2, or 3 sets of chromosomes, or in between) as fermentations progressed to the end (82 h EFT) where the change trend is indicated by the horizontal arrow (bottom overlays)

Figure 3 depicts the cell distribution of non-synchronized cultures at the conditions of 10,000 L scale and DO 25% (dark green) and at 10 L scale with the three DO set points, 5% (orange), 8.5% (magenta), 12.5% (light green), containing 1C, 2C or 3C (vertical arrows), or in between, at 47 h (left-side profiles) and 82 h EFTs (right-side profiles). For comparative purposes to show the cell distribution at 47 h EFT in every experimental condition were superimposed (left-side overlay). Same comparative purpose to show the cell distribution at 82 h EFT in every experimental condition were superimposed (right-side overlay).

Interestingly, the cultures at the 10,000 L when compared with 10 L scale at 47 h EFT; exhibited pronounced differences in cell distribution consisting of larger subpopulations transitioning from 1C to 2C sets of chromosomes. On the other hand, the 3C yeast subpopulation was never apparent in 10 L cultures (Fig. 3).

When comparison was between 47 and 82 h EFT, there were more cells from the 1C subpopulation migrated to the 2C and 3C subpopulations at 10 L scale and every DO set point (horizontal black arrow), including those in transition (between 2C and 3C), as reflected by the area under those peaks. At 10,000 L there was an enhancement of the 2C and 3C subpopulations at 82 h EFT with respect to the 47 h-profile (Fig. 3).

These results suggest that at both scales and every DO set point a large proportion of the yeast cell population had not completed cytokinesis, whereas a relatively smaller subpopulation with 3C had failed to complete both karyokinesis and cytokinesis (Fig. 2), which is in agreement with the hypothesis that a cell cycle arrest has occurred.

### Study of cell morphology distribution

To further characterize the possible presence of mitotic arrest, we analyzed other aspects related to yeast cell size and budding morphology using Vi-CELL and Flow Imaging. Both technologies are based on cell image analysis and vary in the total number cell images analyzed. For Vi-CELL up to 1000 cells per sample in a stagnant phase and flow imaging up to 150,000 cells per sample under constant flow were analyzed.

**Fig. 4:**
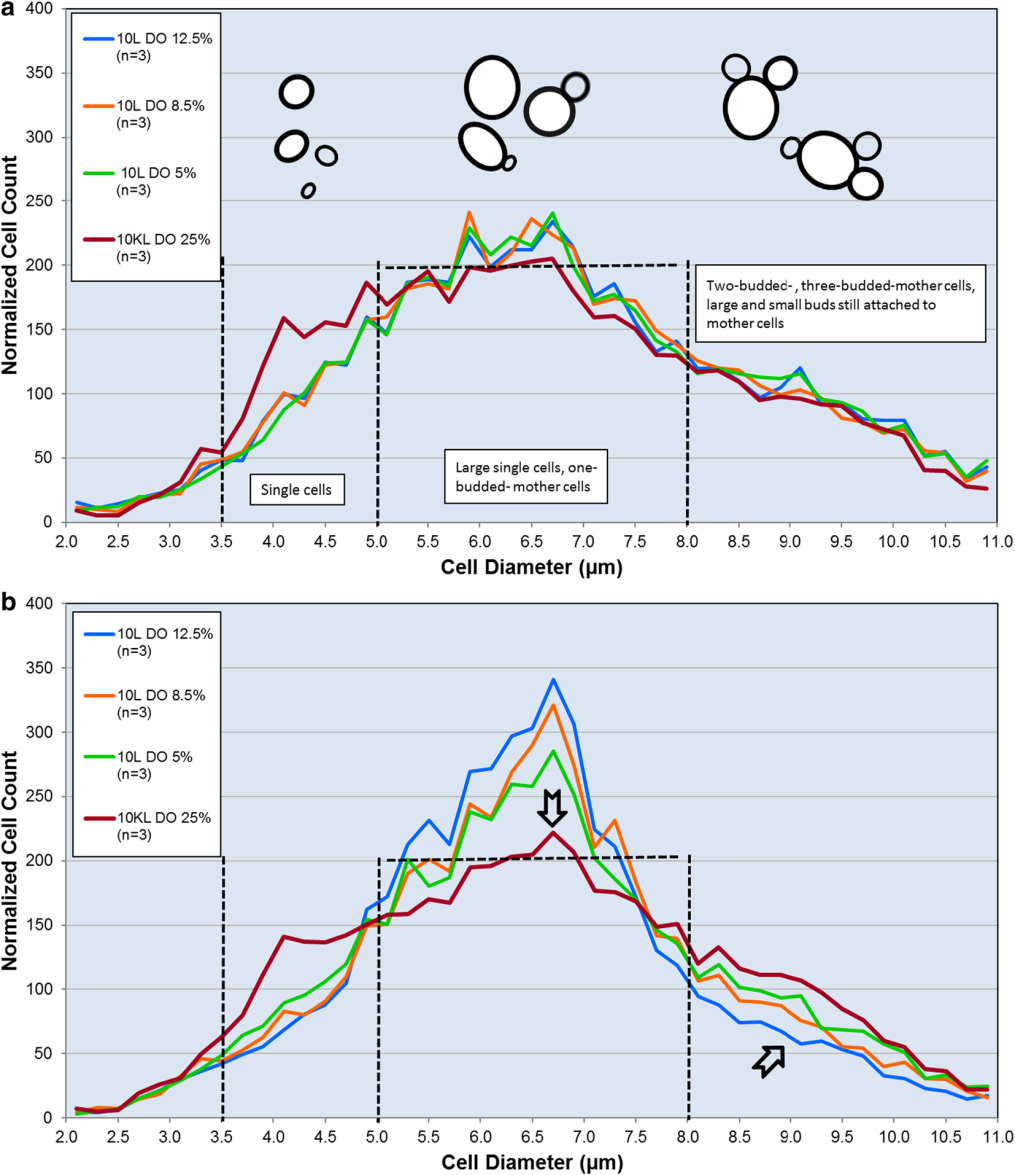
Profiles of the distribution of subpopulations with different cell diameters at elapsed fermentation time 47 h (**a**), and 82 h (**b**). Plotted are the normalized cell counts (NCC) versus the cell diameter from 2 to 11 microns (µm) using Vi-CELL XR Viability Analyzer. Cell count values were normalized by total count for each sample measurement and averaged. Vertical dashed-lines indicate the three ranges of cell diameter, 3.5–5.0, 5.0–8.0, and 8.0–11.0 µm. Above every cell diameter range, the typical associated morphologies observed for those cell sizes. Horizontal dashed-line located at 200 NCC is a reference to assess the relative changes of the subpopulation NCCs as fermentations progressed to the end (82 h EFT) where the change trends are indicated by the arrows

Preliminary experiments allowed us to set up Vi-CELL imaging for identifying any size changes under the experimental conditions detailed in Table 1. Samples were prepared and analyzed as detailed in Methods. Figure 4 shows the plot of average distribution obtained from measurements of normalized cell counts and their diameter (n = 3 per condition). Figure 4a depicts a 47 h EFT normalized distribution of yeast cells as a function of cell diameter. Three cell size ranges corresponding to 3.5–5 μm, 5–8 μm, and 8–11 μm, were characterized and found to be associated with different morphologies within the mitotic cell cycle as also shown in Fig. 4a. Based on Vi-CELL images, the cell morphologies observed within each size range were: 3.5–5 μm for single cell, 5–8 μm for one-budded mother or large mother cell, and 8–11 μm for multi-sized buds as well as one- or two-budded cells.

Except for a slightly higher level of single cells at the 10,000 L scale, at 47 h EFT the distribution of cells in the three size ranges and corresponding budding morphology was similar when compared between scales and at different DOs within samples from the 10 L reactors (Fig. 4a). However, at the 10,000 L scale and at EOR (82 h EFT), we found a decrease in the subpopulation of cells within 5–8 μm range and a concomitant increase of the cell population within the 8–11 μm range when compared to all DO conditions at the 10 L scale, as indicated by the arrows in Fig. 4b. Trending similarly at the 10,000 L scale, the cultures at DO 5% exhibited increasing subpopulations with sizes greater than 8 μm; cell subpopulation within the 3.5–8 μm range displayed the smallest incremental changes by 82 h EFT in comparison with the other two DO levels at the 10 L scale. These observations indicated that low DO produces an enrichment in the cell subpopulation with larger diameter at both scales.

**Fig. 5:**
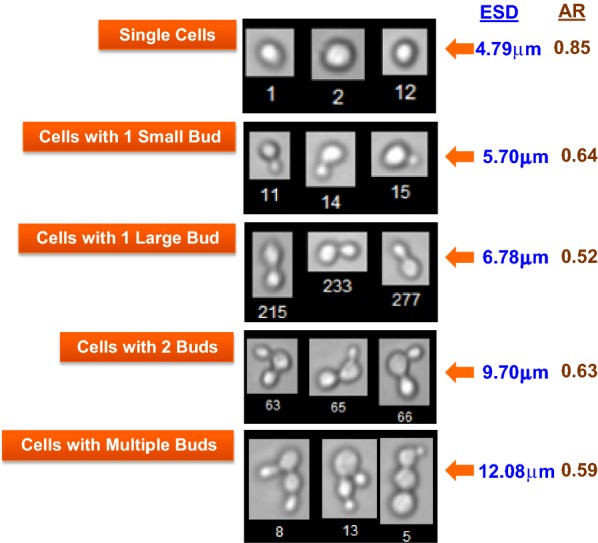
Images of the representative populations of cell morphologies captured using the FlowCAM VS-1 Fluid Imaging Instrument. For the ESD and AR definitions refer to the “Methods” section. The average ESD and AR values are shown for each of the population ranging from 4.79 to 12.08 μm for ESD and from 0.52 to 0.85 for the AR

Flow imaging allowed us to characterize cell morphology and population distribution with higher precision. For example, a more direct quantitative association between cell diameter and cell morphology was obtained based on the Equivalent Spherical Diameter (ESD) and the aspect ratio (AR). Figure 5 illustrates the relationship between the two measurements, showing that AR is the highest in single cells and increases with the number of buds and their relative position to the mother cell, e.g. a more polarized bud positioning in two-budded cells renders a more spherically-shaped cell mirrored by an increase in AR (Fig. 5). In contrast, the grape-shaped multi-budded cells showed low AR and the highest ESD.

**Fig. 6:**
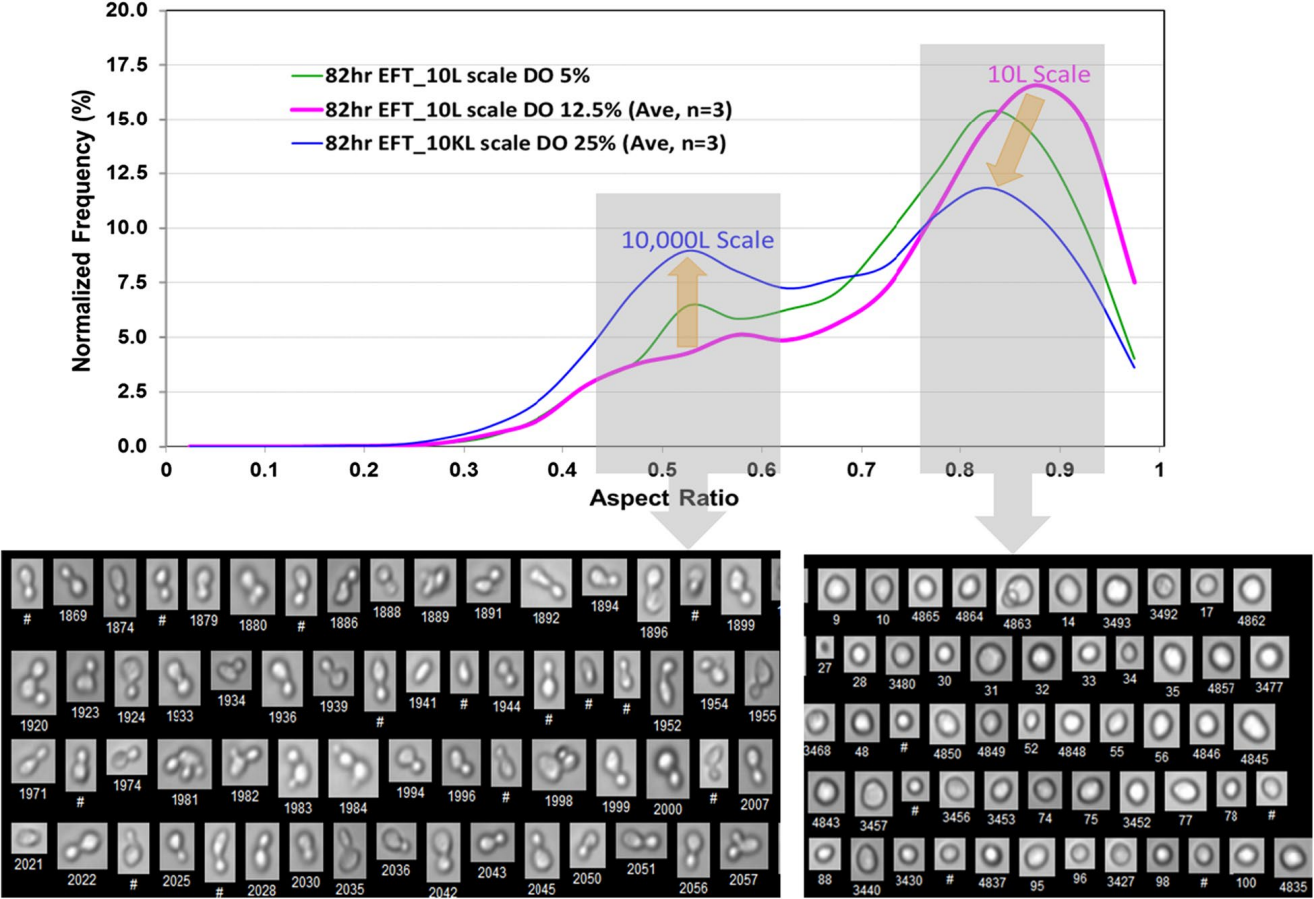
Overlay of cell AR distributions at the end of 10 L and 10 KL bioreactor runs at three different DO set points. Significant differences in distributions attributed to scale and DO levels, are indicated by the arrows and correspond to two main populations with approximate AR values of 0.54 and 0.85. Difference in abundance of populations with ~ 0.5 AR is highlighted in the top panel and respective morphologies of representative cells are shown in the bottom panel. Average AR and ESD values of displayed cells are 0.54 and 7.02 µm respectively. Difference in abundance of populations with ~ 0.9 AR is highlighted in the top panel and respective morphologies of representative cells are shown in the bottom panel. Average AR and ESD values of displayed cells are 0.85 and 5.58 µm respectively

Figure 6 displays the percentage of population at different ARs at EOR (82 h EFT), this time comparing scales between 10,000 L and 10 L, and within 10 L scale at DO set points of 5% and 12.5%. Two frequency peaks corresponding to two cell subpopulations characterized by ARs of approximately 0.54 and 0.85 were present at the 82 h EFT analyzed (Fig. 6). At the 10 L scale, the percentage of the population with AR of 0.85 was more prominent. A population with AR of 0.85 was morphologically comprised by mostly small and large single cells. The data show that in 10 L cultures, a low DO induced an enrichment in the subpopulation characterized by AR of 0.54 which is associated with large-single budded and multi-budded cells exhibiting high *apparent* diameters, concomitant with a drop in the subpopulation having AR of 0.85, approaching the profile found at 10,000 L (indicated by arrows in Fig. 6). The morphology of single-budded cells with large buds, and multi-budded cells with smaller buds, was indicative of incomplete cytokinesis.

In summary, the data presented show that the existence of hypoxia after a 1000-fold scale-up of a bioprocess influences yeast mitosis resulting in cell cycle arrest by impairment of karyokinesis and cytokinesis in a subpopulation of cells. Our hypothesis is that at 10,000 L scale the cells are experiencing hypoxia. When we tried to mimic this at the 10 L scale by reducing the DO set point from 12.5% to 8.5% to 5%, we saw the morphology of the cells approaching that seen at the 10,000 L scale.

## Discussion

The main finding of this study is that hypoxia created by a 1000-fold scale-up of a bioprocess influences yeast mitosis resulting in impairment of cytokinesis and karyokinesis in a subpopulation of cells. This phenomenon happened at the industrial scale and manifested as changes in the distribution of cell size and morphology as a function of time. At the end of the bioreactor run, by 82 h EFT, we detected an increase in cell subpopulations exhibiting characteristic low aspect ratio and large diameter (Figs. 4 and 6). Specifically, cells with larger diameter and lower aspect ratio corresponded to morphologies such as large-single-budded and multi-budded cell populations. Those morphologies became more abundant and are indicative of incomplete cytokinesis at the M phase of *Saccharomyces*’s mitotic cycle.

These morphological alterations can explain the increase in the volume occupied by cells as reflected by WCW measurements [1, 2]. In line with our initial hypothesis of the presence of hypoxic conditions at the end of the industrial scale bioreactor [1] and has been supported by small scale experiments reported herein. At DO set point of 5% in 10 L culture we could approach cell size, aspect ratio, and associated morphological distributions with those observed at the 10,000 L scale. Then these yeast morphological alterations can be explained, at least in part, by hypoxic conditions existing at the 10,000 L scale. Based on the single-cell analysis of *Saccharomyces* cells, the presence of cells with larger diameter were reported during scale-down experiments not directly related to growth reduction but rather to the presence of a zone of strong substrate limitation as well as prolonged exposure to oxygen limitations [16]. Possibly the presence of zones having simultaneously substrate and oxygen limitations could better reproduce the morphological distributions observed at the 10,000 L scale bioreactor.

Hypoxia as a trigger of yeast cell cycle changes resulting in morphological alterations was also supported by previously reported exometabolomics and cell wall integrity data as reflected in the lower resistance to zymolyase [1, 2]. Indeed, the results of the present work agree with our previous data showing partially impaired synthesis of phosphorylated nucleosides which are key intermediates in glycosylation and cell wall synthesis pathways generating chitin, glucans, and poly-mannosylated proteins. Together with the weaker cell wall described by the end of 10,000 L fermentation [2], the present data help explain the origin of high WCWs observed as an enrichment in cell subpopulations of large budded and multi-budded cells, and cell cycle arrest due to cytokinesis impairment.

**Fig. 7:**
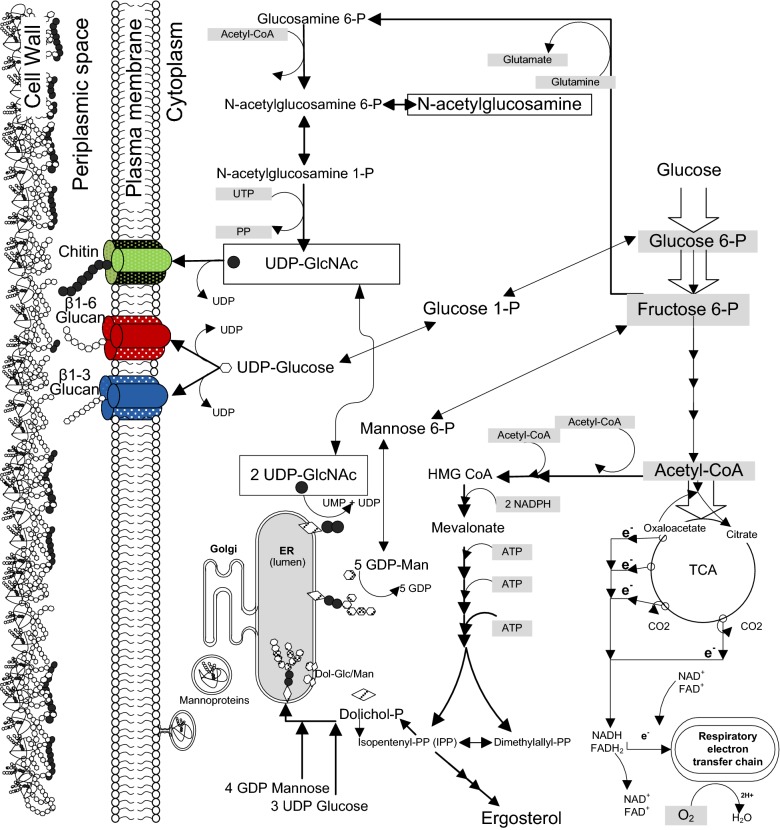
Pathway diagram of phosphorylated nucleosides which are key intermediates in glycosylation and cell wall synthesis pathways generating chitin, glucans, and poly-mannosylated (mannoproteins) proteins. Pathway of *N*-acetyl-glucosamine (GlcNAc) synthesis in cytoplasm from glycolysis, followed by formation of UPD-*N*-acetyl-glucosamine (UDP-GlcNAc) and its polymerization at the plasma membrane complex (green) to form chitin which is translocated into the periplasmic space. The glucans are synthesized from UDP-glucose in the cytoplasm by polymerization at the plasma membrane to form β-1,3- (blue) and β-1,6-glucan (red), and secreted into the periplasmic space. Poly-mannosylated proteins are processed through the endoplasmic reticulum and Golgi for packing in vesicles that are transported to the plasma membrane to be secreted into the periplasmic space

**Fig. 8:**
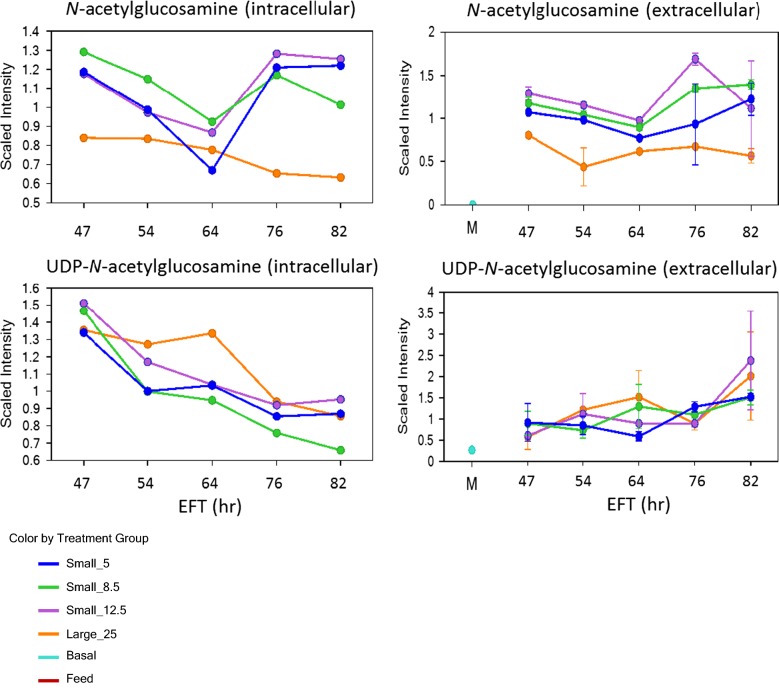
Profiles of *N*-acetyl-glucosamine (GlcNAc) and UDP-GlcNAc relative intracellular and extracellular levels when fermentations are performed at different scales and DO set points. These are key intermediates for chitin synthesis and monitored for differences over time at 10,000 L scale and DO 25% (Large_25) (orange line) and at 10 L scale at DO 5% (Small_5) (blue line), DO 8.5% (Small_8.5) (green line), and DO 12.5% (Small_12.5) (purple line). The intermediates measurement and data analysis were determined as described in “Methods” section. In this line plot graph, data are scaled such that the median value measured across all samples was set to 1.0. Error bars represents “mean +/− one standard deviation”. In the extracellular samples, GlcNAc levels are shown at “M” in the time axis for extracellular medium line plot stand for Basal Medium (light blue), and at Feed (brown) where is undetectable

Chitin is a main component of the septum between mother and daughter, an impairment of its synthesis apparently precluded mother-cell separation as revealed by the presence of multi-budded mother cells [17, 18]. Chitin and glucans (β-1,3- and β-1,6-glucans) are synthesized in the cytoplasm by polymerization of UDP-*N*-acetyl-glucosamine (UDP- GlcNAc) and UDP-glucose, respectively, and secreted into the periplasmic space [19]. Poly-mannosylated proteins are processed through the endoplasmic reticulum and Golgi for packing in vesicles that are transported to the plasma membrane. Figure 7 describes several metabolic pathways known to contribute to protein glycosylation and the synthesis of key polysaccharides for the cell wall formation and their intermediates from these pathways which were monitored for differences over time in the two scales. Glucose-6-phosphate (G6P), fructose-6-phosphate (F6P), mannose-6-phosphate (Man6P), and *N*-acetyl-glucosamine (GlcNAc) were at consistently lower levels in 10,000 L compared to 10 L reactors suggesting impairments in protein glycosylation as well as formation of chitin and glucans [2]. The partially impaired synthesis of glucans was reported to induced multi-budding pattern or pseudo-filaments composed with 5–8 µm cells, no impact on growth and viability, and yeast cells exhibited higher resistance to zymolyase accompanied by an increase in chitin content [20–22]. However, chitin synthesis, likely at the origin of cell cycle arrest at the M phase, is one of the key cellular processes affected by hypoxia [23–25], apparently eliciting a low supply of the key precursor GlcNAc for chitin synthesis throughout the fermentation process (Fig. 8).

Our results expand our previous observations by connecting the metabolic shift induced by hypoxia and the changes found in the yeast’s mitotic cycle, as can be judged from the appearance of subpopulations of large budded and multi-budded cells, suggesting incomplete cytokinesis and thus cell cycle arrest at the M phase (Fig. 2). An impairment of chitin synthesis could, in principle, explain the incomplete cytokinesis at the origin of large single-budded and multi-budded cells.

## Conclusion

In summary, we have characterized an arrest of cell proliferation triggered by hypoxic conditions arising in high cell density yeast cultures at the end of an industrial scale bioreactor run. The results suggest that these conditions lead to a metabolic shift that limits the supply of precursors for chitin synthesis and affects the septum formation at the mother-daughter cells neck precluding cytokinesis.

## Methods

### Strains

The *S. cerevisiae* production strain (PRD) was originally developed from the parental *S. cerevisiae* strain AH22 (ATCC 38626), with the gene encoding the product (Pr-1) in a high-copy plasmid [1]. Host strain was expressing and secreting the Pr-1 molecule under a glucose limiting condition.

## 10 L fermentation process

The details of the 10 L and 10,000 L scale have been reported before [1]. Briefly, the seed train started in a shake flask incubated and agitated in a rotary shaker at 30 °C and 225 rpm until glucose was depleted. Then the flask culture was used to inoculate a seed bioreactor (Biolafitte, Saint Germain en Laye, France) to achieve higher cell density with the following set points: backpressure 10 psi, dissolve oxygen (DO) 30%, pH 6.0 and temperature 29 °C. This seed stage consisted of a batch phase followed by a fed-batch phase using glucose nutrient feed. The pH and DO control loops were the same as described before via Distributed Control System (DCS, Siemens Moore APACS, USA) [1]. DO was controlled at a cascade system of automatically first ramping up agitation until the maximum (900 rpm), and then injecting pure oxygen being blend into the inflowing air fixed at a total gas sparge rate of 10 slpm, if needed. The pH was controlled using ammonium hydroxide (30%) and phosphoric acid (17%) additions. Once the target optical density (OD_600_) of 100 ± 20 was reached, the culture was used to inoculate a 10 L production bioreactor with a target inoculation of OD_600_ 15. The production process was set at a backpressure of 10 psi, pH of 5.75, temperature of 29 °C, and DO at 12.5%, 8.5%, or 5%. A glucose nutrient feed and a phosphate salt solution feed were both initiated when glucose concentration was ≤ 0.3 g/L in the batch medium. The shake flask medium (modified buffered minimal medium (BMM)), seed media (modified MW10 medium) and feed media (modified MW10 medium) were the same as described in Fu et al. [1]. Samples were taken at certain intervals to monitor biomass by measuring OD_600_, WCW, and DCW. The fermentation process was terminated after 82 h EFT. At pre-determined time points, fermentation supernatants and pellet samples were collected for metabolite profiling.

### 10,000 L fermentation process

A full seed train was implemented, including 2 stages of shake flask cultures and 2 stages of seed bioreactors. The scale-dependent process parameters such as batch volume, glucose nutrient and phosphate salt feed rates as well as total gas flow rate were accordingly scaled-up as detailed in Fu et al. [1]. At pre-determined time points, fermentation supernatants and pellet samples were collected for metabolite profiling.

### Wet cell weight and cell dry weight determinations

At periodic intervals, samples were taken for determination of wet cell weight (WCW) and cell dry cell weight (DCW). The details of WCW and CDW determinations are detailed in Fu et al. [1].

### Cell viability and DNA content by flow cytometry

The *S. cerevisiae* cell viability was measured using a BD FACS Calibur Flow Cytometer (BD Bioscience, San Jose, CA, USA) with some modifications [1]. Samples taken from the culture were immediately diluted with phosphate buffered saline (PBS; pH 7.2) to a final optical density at 600 nm (OD_600_) of 1 using a Beckman coulter model DU720 spectrophotometer (Beckman Coulter, Inc., Brea, CA, USA). Later stained with the red fluorescence dye, propidium iodide (PI) stock solution 1 mg/mL (Sigma-Aldrich, catalog #: P4864), by adding 16 μL/mL of cell suspension. The fluorophore only stains the DNA of nonviable cells. The cell viability was reported as the inverse of the percentage of PI-positive cells with respect to the total number of cells.

Using the same fluorophore PI to measure DNA content, cells were treated based on the following description. Samples of broth were diluted to reach an OD_600_ of 1 using PBS solution. Sample was centrifuged and the cell pellet resuspended in 1 mL of 70% ethanol. After a fixation period of at least a 1 h, the cell suspension was centrifuged for 1 min at 13,600 rpm, and the cell pellet resuspended in 1 mL of 50 mM sodium citrate (pH 7). The cell suspension was sonicated for 15 secs, centrifuged for 6 min at 10,000×*g*, and the cell lysate pellet resuspended in 1 mL of sodium citrate (pH 7). RNase A stock solution (10 mg/mL) (Fisher Scientific, catalog #: FEREN0531) was then added to a final concentration of 0.25 mg/mL to remove RNA background interference. The suspension was incubated at 50 °C for 1 h or overnight at 37 °C. The resulting cell suspension was centrifuged for 6 min at 10,000×*g* and the pellet was washed using sodium citrate (pH 7). This wash step was performed twice. Staining was performed by adding 16 µL of PI stock solution (1 mg/mL) into 1 mL of cell suspension. The prepared sample was then processed using flow cytometry in which a minimum of 10,000 cells were counted per sample. Sample processing and analysis was performed using a Macintosh Quadra 650 running the Cell Quest Pro software. The relative standard error (RSD) between different analysis of the same sample was only 1 to 2%.

### Cell morphology characterization based Vi-CELL technology

#### Measurement of viable cell concentration and cell size

Viable cell concentration was determined by the trypan blue exclusion method [26] using a Vi-CELL XR Viability Analyzer (Beckman Coulter, Inc., Brea, CA, USA). Yeast samples were analyzed using a modified *Saccharomyces* yeast cell type that optimized yeast images for the production process as described in the Particle Characterization, *Analyzing Yeast Using the Vi*-*CELL™ XR* 2014 (Beckman Coulter, Inc., Brea, CA, USA) documentation.

The following parameter settings were applied: Minimum diameter (µm) of 2; Maximum diameter (µm) of 11; Number of images of 50; Aspirate cycles of 5; Trypan blue mixing cycles of 9; Cell brightness (%) of 85; Cell sharpness of 100; Viable cell spot brightness (%) of 40; Viable cell spot area (%) of 1; Minimum circularity of 0.65; and Decluster degree of High. Yeast time-course samples were diluted accordingly with 1X PBS solution to ensure that raw total cell concentration (TCC) did not exceed 10 × 10^6^ cells per mL, the upper limit for cell counts. Samples, however, were diluted at around 200-fold to maintain cell counts below 1000 per image, a requirement for processing supplemental images using an orthogonal microscopic method.

### Cell size and morphology characterization based on flow imaging

A FlowCAM VS-1 Fluid Imaging Instrument (Fluid Imaging Technologies Inc., Scarborough, ME) fitted with 20× objective was used for cell imaging analysis. The analysis was performed using FC100 flow cells. Focusing was performed using 10 microns (μm) polystyrene beads (4210A, ThermoFisher Scientific) and the automated routine in the VisualSpreadsheet software package. Cell Imaging analysis was performed using both live and fixed yeast cells. Between samples the flow cell was cleaned using 10% Contrad 70 (Decon Laboratories, Inc.) liquid detergent solution and particle free water. The fixed cell samples were prepared by 40-fold dilution of the culture broth in 70% (v/v) Ethanol, after which the samples were stored at 2–8 °C. Prior to analysis cell suspensions (both fixed and live) were homogenized by sonication in amplitude 30% continuous mode with 2 cycles of 10 secs using Sonifier model SLPt (Branson Ultrasonics Corporation, Danbury, CT, USA). After each sonication cycle, samples were chilled in a wet ice bath for 60 secs. Finally, the homogenized cell suspensions were 400-fold diluted with PBS buffer and equilibrated to room temperature. The dilution is to allow the analysis of at least 150,000 cells per sample. Prior to the data analysis, low resolution images were removed by applying the edge gradient filter (30 to 255). The duplicate and partial images were also removed manually.

Statistical calculations and data overlays were performed in Microsoft Excel using imported values from VisualSpreadsheet report. Prior to overlaying and comparing data, the Frequency values were normalized by total count for each sample measurement and averaged were appropriate.

There are two measurements tracked statistically for every cell, one is the equivalent spherical diameter (ESD) in microns (µm), and the aspect ratio (AR). ESD is the mean value of 36 feret measurements. Feret measurement is defined as the perpendicular distance between parallel tangents touching opposite sides of the cell. VisualSpreadsheet makes 36 measurements for each cell, one each 5 degrees between − 90 degrees and + 90 degrees. AR is the ratio of the length of the minor axis over the length of the major axis, so this is a non-dimensional measurement.

### Metabolite profiling

#### Study design

Samples were obtained from three batches at 10,000 L scale, and at 10 L scale triplicates at every DO set point tested, 12.5%, 8.5%, and 5%. A total of five media replicates (or spent media) for each scale were collected at EFT 46.5, 54.0, 63.5, 76.0, 81.5, and frozen at − 80 °C, and later analyzed for metabolite profiling at Metabolon, Inc. (Durham, NC, US). Additionally, one batch medium sample and one glucose feed sample were included for reference.

### Sample preparation

Spent medium samples were thawed on ice, and a 100 μL volume sample was used for extraction. Samples were prepared for the appropriate instrument, either liquid chromatography/mass spectrometry/ mass spectrometry (LC/MS/MS) or gas chromatography/mass spectrometry (GC/MS) as described in Fu et al. [1].

### Liquid chromatography/mass spectrometry and gas chromatography/mass spectrometry

The LC/MS portion of the platform incorporated a Waters Acquity UHPLC system and a Thermo-Finnigan LTQ mass spectrometer, including an electrospray ionization source and linear ion-trap mass analyzer were used to analyze all the samples. Aliquots of samples which were vacuum-dried, reconstituted; and run through the different analytical chromatographic columns are detailed in Fu et al. [1].

### Compound identification, quantification, and data curation

Metabolites were identified by automated comparison of the ion features in the experimental samples to a reference library of over 3000 purified, authenticated chemical standard entries that include retention time, molecular weight (m/z), preferred adducts, and in-source fragments as well as their associated MS/MS^2^ spectra. In brief, samples were extracted and split into equal parts for analysis on the GC/MS and two LC/MS/MS platforms. Proprietary software was used to match ions to the in-house library of standards for metabolite identification and for metabolite quantization by peak area integration.

### Data collection, normalization and visualization

A client matrix composed of small aliquots of all sample extracts in this study was created. The client matrix technical replicate samples were treated independently throughout the process. All process samples (client matrix, and a mixture of organic components used to assess GC column performance, process blanks, etc.) were spaced evenly among the injections for each day and all samples were randomly distributed throughout each day’s run. Data were collected over multiple platform run days and thus, ‘block normalized’ by calculating the median values for each run-day block for each individual compound. This minimizes any inter-day instrument gain or drift, but does not interfere with intra-day sample variability. Missing values (if any) were assumed to be below the level of detection for that biochemical with the instrumentation used and were imputed with the observed minimum for that particular biochemical.

For visualization of biochemical differences between the various treatment groups, the data are displayed in line plot graph format and scaled such that the median value measured across all samples set to 1.0. The data selected for display by line plot were filtered by statistics or included for completion of a biochemical pathway.

## Abbreviations

DCS: distributed control system
DO: dissolved oxygen
WCW: wet cell weight
DCW: dry cell weight
EOR: end of run
EFT: elapsed fermentation time
10 KL_Reactor: 10,000 L reactor
Ave: average
ESD: equivalent spherical diameter
AR: aspect ratio
PI: propidium iodide
ER: endoplasmic reticulum
IPP: isopentenyl-pyrophosphate
DolP: dolichol-phosphate
GlcNAc: *N*-acetyl-glucosamine
HMG-CoA: 3-hydroxy-3-methly-glutaryl-CoA
CoA: coenzyme A
UDP: uridine-diphosphate
GDP: guanidine-diphosphate
ATP: adenosine-triphosphate
NAD^+^: nicotinamide adenosine dinucleotide
FAD^+^: flavin adenine dinucleotide
NADH/FADH: reduced forms of NAD^+^/FAD^+^
LIT: linear ion-trap
slpm: standard liter per minute
psi: pound per square inch
TCA: tricarboxylic acid cycle
PR: production
L: liter
GC/MS: chromatography/mass spectrometry

## References

[1] Fu Z, Verderame TD, Leighton JM, Sampey BP, Appelbaum ER, Patel PS, Aon JC (2014). Exometabolome analysis reveals hypoxia at the up-scaling of a *Saccharomyces cerevisiae* high-cell density fed-batch biopharmaceutical process. Microb Cell Fact.

[2] Aon JC, Sun J, Leighton J, Appelbaum E (2016). Hypoxia-elicited impairment of cell wall integrity, glycosylation precursors synthesis, and growth in scaled-up high-cell density fed-batch cultures of *Saccharomyces cerevisiae*. Microb Cell Fact.

[3] Cid VJ, Duran A, Del Rey F, Snyder MP, Nombela C, Sanchez M (1995). Molecular basis of cell integrity and morphogenesis in *Saccharomyces cerevisiae*. Microbiol Rev.

[4] Biely P (1978). Changes in the rate of synthesis of wall polysaccharides during the cell cycle of yeast. Arch Microbiol.

[5] Díaz S, Zinker S, Ruiz-Herrera J (1992). Alterations in the cell wall of *Saccharomyces cerevisiae* induced by the alpha sex factor or a mutation in the cell cycle. Antonie Leeuwenhoek.

[6] De Nobel JG, Klis FM, Ram A, Van Unen H, Priem J, Munnik T, van den Ende H (1991). Cyclic variations in the permeability of the cell wall of *Saccharomyces cerevisiae*. Yeast.

[7] Bhavsar-Jog YP, Bi E (2017). Mechanics and regulation of cytokinesis in budding yeast. Semin Cell Dev Biol.

[8] Rodriguez-Pena JM, Cid VJ, Arroyo J, Nombela C (2000). A novel family of cell wall-related proteins regulated differently during the yeast life cycle. Mol Cell Biol.

[9] Wauters T, Iserentant D, Verachtert H (2001). Impact of mitochondrial activity on the cell wall composition and on the resistance to tannic acid in *Saccharomyces cerevisiae*. J Gen Appl Microbiol.

[10] Aguilar-Uscanga B, Francois JM (2003). A study of the yeast cell wall composition and structure in response to growth conditions and mode of cultivation. Lett Appl Microbiol.

[11] Blanco N, Reidy M, Arroyo J, Cabib E (2012). Crosslinks in the cell wall of budding yeast control morphogenesis at the mother-bud neck. J Cell Sci.

[12] Shaw JA, Mol PC, Bowers B, Silverman SJ, Valdivieso MH, Duran A, Cabib E (1991). The function of chitin synthases 2 and 3 in the *Saccharomyces cerevisiae* cell cycle. J Cell Biol.

[13] Fu Z, Leighton J, Cheng A, Appelbaum E, Aon JC (2012). Optimization of a *Saccharomyces cerevisiae* fermentation process for production of a therapeutic recombinant protein using a multivariate Bayesian approach. Biotechnol Prog.

[14] Fu Z, Baker D, Cheng A, Leighton J, Appelbaum E, Aon JC (2016). Characterization of a *Saccharomyces cerevisiae* fermentation process for the production of a therapeutic recombinant protein using multivariate Bayesian approach. Biotechnol Prog.

[15] Hartwell LH, Unger MW (1977). Unequal division in *Saccharomyces cerevisiae* and its implications for the control of cell division. J Cell Biol.

[16] Marba-Ardebol AM, Bockisch A, Neubauer P, Junne S (2018). Sterol synthesis and cell size distribution under oscillatory growth conditions in *Saccharomyces cerevisiae* scale-down cultivations. Yeast.

[17] Klis FM, Mol P, Hellingwerf K, Brul S (2002). Dynamics of cell wall structure in *Saccharomyces cerevisiae*. FEMS Microbiol Rev.

[18] Popolo L, Gilardelli D, Bonfante P, Vai M (1997). Increase in chitin as an essential response to defects in assembly of cell wall polymers in the ggp1 mutant of *Saccharomyces cerevisiae*. J Bacteriol.

[19] Cabib E, Arroyo J (2013). How carbohydrates sculpt cells: chemical control of morphogenesis in the yeast cell wall. Nat Rev.

[20] Hong Z, Mann P, Brown NH, Tran LE, Shaw KJ, Hare RS, DiDomenico B (1994). Cloning and characterization of KNR4, a yeast gene involved in (1,3)-beta-glucan synthesis. Mol Cell Biol.

[21] Daran JM, Dallies N, Thines-Sempoux D, Paquet V, Francois J (1995). Genetic and biochemical characterization of the UPG1 gene encoding the UDP-glucose pyrophosphorylase from *Saccharomyces cerevisiae*. Eur J Biochem.

[22] Daran JM, Bell W, Francois J (1997). Physiological and morphological effects of genetic alterations leading to a reduced synthesis of UDP-glucose in *Saccharomyces cerevisiae*. FEMS Microbiol Lett.

[23] Cid VJ, Adamikova L, Cenamor R, Molina M, Sanchez M, Nombela C (1998). Cell integrity and morphogenesis in a budding yeast septin mutant. Microbiology.

[24] Igual JC, Johnson AL, Johnston LH (1996). Coordinated regulation of gene expression by the cell cycle transcription factor SWI4 and the protein kinase C MAP kinase pathway for yeast cell integrity. EMBO J.

[25] Schmidt M, Bowers B, Varma A, Roh DH, Cabib E (2002). In budding yeast, contraction of the actomyosin ring and formation of the primary septum at cytokinesis depend on each other. J Cell Sci.

[26] Tennant J (1964). Evaluation of trypan blue techniques for determination of cell viability. Transplantation.

